# A Gecko-Inspired Robot with a Flexible Spine Driven by Shape Memory Alloy Springs

**DOI:** 10.1089/soro.2022.0080

**Published:** 2023-08-09

**Authors:** Jiahui Qiu, Aihong Ji, Kongjun Zhu, Qinfei Han, Wei Wang, Qian Qi, Guangming Chen

**Affiliations:** ^1^ Lab of Locomotion Bioinspiration and Intelligent Robots, College of Mechanical and Electrical Engineering, and College of Aerospace Engineering, Nanjing University of Aeronautics and Astronautics, Nanjing, China.; ^2^ State Key Laboratory of Mechanics and Control of Mechanical Structures, College of Aerospace Engineering, Nanjing University of Aeronautics and Astronautics, Nanjing, China.

**Keywords:** gecko robot, lateral swing pattern, flexible spine, SMA spring, kinematics

## Abstract

The majority of sprawling-posture quadrupedal vertebrates, such as geckos and lizards, adopt a cyclical lateral swing pattern of their trunk that is coordinated with limb movements to provide extraordinary flexibility and mobility. Inspired by the gecko's locomotory gait and posture, a gecko-like robot with a flexible spine driven by shape memory alloy (SMA) springs was proposed in this work. The static parameters of the SMA spring were experimentally measured, and the flexible spine driven by SMA springs can be deflected bidirectionally. A kinematic model of the spine mechanism was established, and the mathematical relationship between the thermodynamic behavior of the SMA springs and spinal deflection was systematically analyzed. When a gecko trots with a lateral swing pattern of its trunk, the body and the spine show a standing wave shape and a single-peak C-type curve, respectively. The lateral spine deflection and trotting gait were combined in a collaborative model of a flexible spine and limbs to describe in detail the specific relationships between leg joint variables and spine deflection angle. Planar motion tests of a prototype robot were also conducted by using four high-speed cameras to record the trajectory of each point of the body, which verified the proposed model. From the acquired results, it was demonstrated that, compared with a rigid body, a robot with a flexible spine has a longer stride length, higher speed, and a greatly reduced turning radius.

## Introduction

A flexible body plays a quite important role in the movement of prostrate quadruped reptiles.^1,2^ These animals, such as geckos and lizards, adopt a cyclical lateral swing pattern of their trunk during movement to gain a locomotory advantage.^3–5^ Dai and Zaaf's work shows that geckos use periodic lateral bending of the spine to coordinate with the swinging of the limbs to achieve a greater range of motion.^6–8^ Cabelguen *et al.* also suggested that the gecko's flexible spine serves two important roles in climbing: (1) counteracting the twisting force from the limbs to the trunk and (2) creating an upward impact force.^9^ Various reports in the literature have also shown that a flexible spine with multiple degrees of freedom allows animals to change direction with a small turning radius.^10^ As a result, coordinated with the limb function, a flexible spine can minimize joint torque and give animals an unusually agile movement.^11^

Currently, the majority of works in the literature on gecko-inspired robots have used structures with a rigid torso. The main focus lies on the development of robots with the gecko's foot structure, which exhibits extraordinary adhesion and desorption mechanisms and spatial transition ability.^12–16^ However, since rigid body structures are not compliant, the body posture has difficulty in achieving dynamic adjustment.^17^ As a consequence, there is a large disparity in the locomotory performance of gecko robots and real geckos.

Typically, flexion and extension processes in vertebrates mainly rely on bones and their surrounding muscles.^18^ In the current research on flexible spines, the actuator is mainly driven by a motor, pneumatic fluid, or rope.^19–21^ Although these driving modes give the robot certain flexibility, additional mechanical devices are also required, resulting in an increase in the overall weight and energy consumption of the robot.^22^ Actuators based on flexible intelligent materials are lightweight and can produce motion deformation under external excitation, such as from light and heat. Therefore, the driving structure can give mechanical feedback to external changes.^23–25^ Among such types of materials, shape memory alloys (SMAs) are particularly attractive because of their simple drive mode, noiseless operation, and high energy density.^26–29^ Due to these characteristics, various forms of SMA have been widely used in the field of biological bionics to simulate biological muscle structures in recent years.^30–34^

More specifically, Kim *et al.* developed a bionic turtle driven by an SMA wire, which simulated the forward and backward torsion of turtle fins by changing the angle between the SMA wire and the support structure.^31^ Yan *et al.* proposed a flexible pectoral fin based on an SMA plate that was capable of performing the three-dimensional (3D) motion.^32^ Mohanakrishnan and Iyamperumal developed an underwater bionic robotic fish by using an SMA spring and lightweight 3D-printed components, which can swim stably in water at a speed of 24.5 mm/s.^34^ However, there are currently no reports in the literature on gecko-like robots with flexible spines driven by SMAs.

Along these lines, in this work, a preliminary investigation into the SMA spring was conducted, whereas its driving force and deformation displacement were experimentally determined. A bionic flexible spine with a multisection hinge structure driven by SMA springs was also designed. The output characteristics of the SMA springs were used to drive the flexible spine to achieve independent deflection without requiring any additional mechanical devices. The input power was varied by using pulse width modulation (PWM) signals with different duty cycles to adjust the joule heating temperature to control the flexible spine deflection angle. A planar kinematic model of the flexible spine system was also derived to describe the motion behavior.

Based on research on the morphology and kinematics of geckos, a collaborative model of the flexible spine and limbs was then established. The kinematic relationship between the deflection angle of the spine and the joint angle of limbs was thoroughly explored. Finally, a gecko-like robot prototype was tested in straight-line and turning crawling experiments to verify its performance.

## Materials and Methods

### SMA spring actuator

There are many forms of SMA, among which the SMA wires and SMA springs are most widely used.^35^ However, although SMA wires can provide relatively large output forces, their strain is limited and is insufficient to produce a considerable deflection angle of a spinal structure.^36^ In contrast, SMA springs sacrifice some output force but can provide a sufficient driving stroke for this application.^26^

**FIG. 1.**
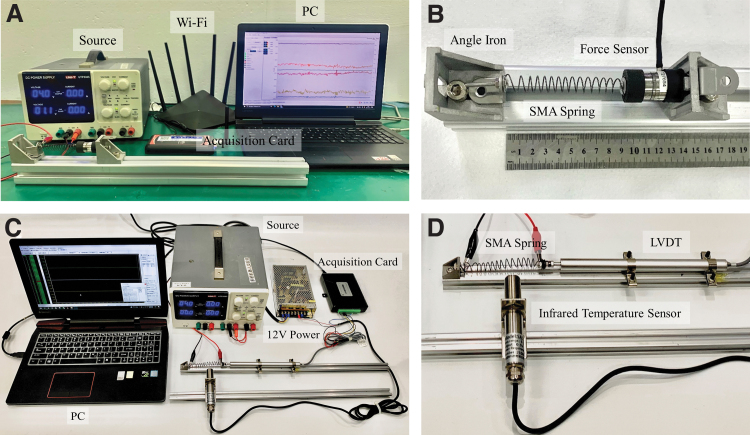
Experiments of the SMA spring. **(A)** Experimental device of fixed-length heating. A mobile power supply provides power to the electric heating drive of the SMA spring. A six-dimensional force sensor (FT27654; ATI Industrial Automation) measures the axial tension generated when the SMA spring is heated, and the tension data are transmitted to a computer through Wi-Fi. **(B)** One end of the SMA spring is fixed on the angle iron, and the other is connected to a relay sensor to stretch the spring to different lengths and measure the output forces generated when it is heated. **(C)** Experimental device that was used to measure the relationship between the SMA spring shrinkage deformation and the heating temperature. The mobile power supply provides power for heating the SMA spring. The data are transmitted to a computer through an acquisition card. **(D)** The temperature of the SMA spring was measured by an infrared sensor. The shrinkage variable was measured by a 12 V displacement sensor (Linear Variable Differential Transformer). SMA, shape memory alloy.

**FIG. 2.**
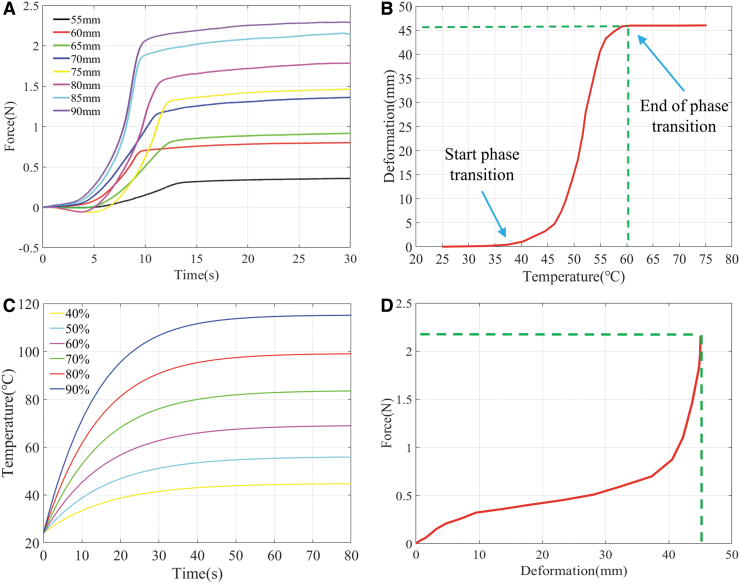
**(A)** Axial force outputs over time by phase transformation of an SMA spring at different tensile lengths. **(B)** Relationship between the SMA spring shrinkage deformation and the temperature. **(C)** Temperature increases with time in the SMA spring under PWM signals with different duty cycles. The input voltage was 5 V at the 100% duty cycle. **(D)** Relationship between the axial force output and the shrinkage deformation of the SMA spring. PWM, pulse width modulation.

Ni-Ti SMA springs were used in this work (JiangYin FaSeng Pell New Material Technology Co. Ltd.) with a Ni content of 50.8%. The experiments were carried out to understand its thermomechanical behavior. Figure 1 illustrates the experimental SMA spring fixed-length heating device. The initial length of the SMA spring was 40 mm. In our previously reported work, it was found that when the fixed length was set below the value of 55 mm, almost no obvious axial output force was produced. Hence, it is believed that this selection has no practical reference significance for the robot. Consequently, the fixed length of the SMA spring started at the value of 55 mm and gradually increased in 5 mm intervals. The SMA spring was electrified and heated within a certain length. The tension changes in the SMA spring at different fixed lengths are shown in Figure 2A.

Obviously, the tension variations in the SMA spring at different lengths were basically the same, while the maximum tension increased as the fixed length became longer. However, when the fixed length increased from 85 to 90 mm, the maximum tensile force only increased from 2.16 to 2.29 N, and the increase rate of tensile force was almost unchanged. In fact, the application of a longer tensile length of the SMA spring leads to a greater shear strain inside it. In this state, the electric heating of the spring accelerates the failure of the shape memory element.

Therefore, it can be argued that if the SMA spring is integrated into the robot with an excessive tensile length, it will not only fail to bring about an effective performance improvement, but also the service life of the robot will be significantly reduced. Consequently, after comprehensive consideration, 85 mm was selected as the tensile length of the SMA spring.

As can be seen from the abovementioned results, this SMA spring can meet the driving requirements of the flexible spine, in terms of load capacity. On the contrary, the specific relationship between the contraction shape variable generated by the SMA spring and the heating temperature must be determined in advance. Therefore, a deformation-temperature measurement experimental device was built (Fig. 1C, D). Figure 2B depicts the relationship between the SMA spring's shrinkage deformation and the local temperature distribution. Under joule heating, the SMA spring's shrinkage deformation continued to increase but then gradually saturated. More specifically, the SMA spring started to contract at about 40°C and the shrinkage deformation was stabilized at about 60°C. The total shrinkage shape variable that was produced in this process was 45 mm, which theoretically meets the driving stroke requirement for the SMA spring to drive the flexible spine.

Based on the abovementioned analysis, by controlling the heating temperature of the SMA springs on both sides of the flexible spine, the degree of contraction can be directly adjusted. Then, the flexible spine can be deflected at any angle in the workspace. The change in the temperature depends on the input voltage. However, the voltage amplitude of the power source was constant. The PWM technique uses a discrete digital level signal to simulate a continuously changing analog signal level.^37^ The voltage amplitude can be adjusted by changing the duty cycle of the PWM signal. More specifically, the relationship between the SMA spring temperature T(°C) and the PWM signal duty cycle P(%) can be described by using the following differential equation^38^:

(1) $$\frac{P^{2}U^{2}}{R}-hA_{s}\left(T-T_{0}\right)=MC_{p}\frac{dT}{dt},$$

where U (V) denotes the constant voltage, t (s) represents the heating time, $T_{0}$(°C) stands for the initial temperature, M (kg) refers to the spring mass, R(Ω) is the spring resistance, $C_{p}$(J/°C·kg) denotes specific heat capacity, h(W/°C·m^2^) signifies the heat transfer coefficient, and $A_{s}$(m^2^) is the heat-exchange area, which can be written as follows^38^:

(2) $$A_{s}=N\pi^{2}dD+\frac{\pi d^{2}}{4},$$

where *N* denotes the number of coils, d(mm) represents the diameter of the spring wire, and D(mm) refers to the coil diameter of the spring. A five constant-voltage power supply was used here. The corresponding curves of the temperature increase with time of the SMA spring under PWM signals with different duty cycles are shown in Figure 2C. By considering both time and temperature as related variables, the relationship between the axial force output and the shrinkage deformation of the SMA spring can be obtained, as can be observed from Figure 2D.

### Mechanism design

Haomachai *et al.* used the least-squares method to model a gecko's spine joints, and the spine of the robot that was driven by a servo motor.^16^ If the active rotation of the motor is not considered, the position of the motor output shaft can be approximately regarded as a rotating joint. In addition, the connecting piece between each of the two motors represents a connecting rod. This is consistent with the flexible spine described in this work, in terms of the motion principle. The approximate exponential convergence of the total error value shows that three joints provide a good trade-off between the geometric structure and the overall length and weight of the robot.^16^ In this work, a bionic flexible spine was designed with three degrees of freedom of rotation, which was composed of three identical submodules. Each submodule was provided with a revolute joint.

**FIG. 3.**
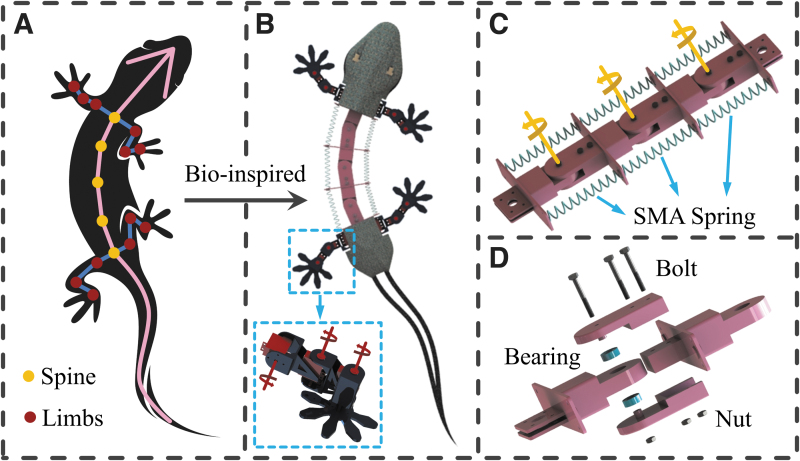
Gecko-inspired mechanism design. **(A)** The body structure of a real gecko. **(B)** Integrated application of a flexible spine in a gecko-like robot, using a limb mechanism with three degrees of freedom. **(C)** The bionic flexible spine with a multisegment hinge structure driven by three pairs of SMA springs. **(D)** Exploded view of a single segment of the flexible spine.

Figure 3C displays the overall structural design of the flexible spine, which adopted a differential design on each side. A hinge structure is formed that can rotate freely in the horizontal plane, and the structural stiffness in the vertical direction is ensured (Fig. 3D).

In addition, the flexible spine is used in a gecko-inspired robot. The structural design and the geometric proportions of the robot are very similar to those of a real gecko, as can be ascertained from Figure 3A and B. To ensure that the robot can realize free 3D single-limb movement without redundant drive, for each leg, a design with three motion joints was used (Fig. 3B), which respectively, correspond to the hip, knee, and ankle joints.

## Kinematic Characteristic

### Flexible spine kinematics

**FIG. 4.**
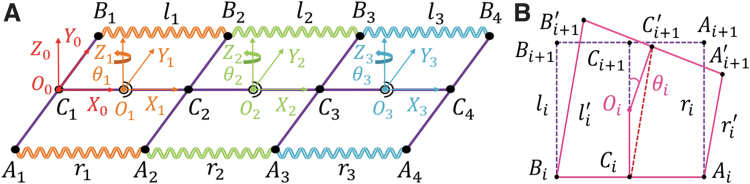
Kinematic model of the flexible spine. **(A)** Plane geometric frame model of the flexible spine. **(B)** Deflection motion characteristics and geometric relationship of each submodule.

To simplify the analysis, the flexible spine was simplified into four links and a plane geometric frame model was established (Fig. 4A). This model was used to determine the mathematical relationship between the shrinkage variable of the SMA springs and the deflection angle of the flexible spine. Figure 4A describes the definitions of each coordinate system and the physical quantity. *A_i_* and $B_{i}\left(i=1,2,3,4\right)$ denote the connection point between the SMA springs and spine frame, *C_i_* stands for the fixed node on the spinal line, where $A_{i}C_{i}=B_{i}C_{i}=m$, and $C_{i}C_{i+1}=2n$. Moreover, $\left\{O_{0}\right\}$ *defines the fixed coordinate system on the spine, where the coordinate axis is expressed as follows* $\left(X_{0},Y_{0},Z_{0}\right)$, while $\left\{O_{j}\right\}\left(j=1,2,3\right)$ defines the coordinate system of each rotational center, with the coordinate axis expressed as $\left(X_{j},Y_{j},Z_{j}\right)$. *l_j_* and *r_j_* represent the initial lengths of the SMA springs on both sides, while $\theta_{j}$ refers to the rotation angle of each rotating joint, with the clockwise direction being positive.

Since the deflection characteristics of each submodule are similar, one of the submodules was taken for analysis (Fig. 4B). The initial state of the module is shown by the dotted line. When the SMA spring on one side (taking the right side as an example) produced shrinkage deformation, the lengths of the SMA springs on both sides were changed to $r_{j}^{\prime }$ and $l_{j}^{\prime }$, respectively, while the shape variables were $\Delta r_{j}$ and $\Delta l_{j}$. At this time, the frame was deflected $\theta_{j}$ to the right and the module changed to the state shown by the solid line. As can be seen from Figure 4B, the SMA spring length and the other structural parameters were related by the following equation:

(3) $$\left\{\begin{array}{c} r_{j}^{\prime }=2n\cos\frac{\theta_{j}}{2}-2m\sin\frac{\theta_{j}}{2}\\ l_{j}^{\prime }=2n\cos\frac{\theta_{j}}{2}+2m\sin\frac{\theta_{j}}{2} \end{array}\right.\left(j=1,2,3\right),$$

(4) $$\left\{\begin{array}{c} \Delta r_{j}=r_{j}^{\prime }-r_{j}=2n\left(\cos\frac{\theta_{j}}{2}-1\right)-2m\sin\frac{\theta_{j}}{2}\\ \Delta l_{j}=l_{j}^{\prime }-l_{j}=2n\left(\cos\frac{\theta_{j}}{2}-1\right)+2m\sin\frac{\theta_{j}}{2} \end{array}\right.\left(j=1,2,3\right).$$

**FIG. 5.**
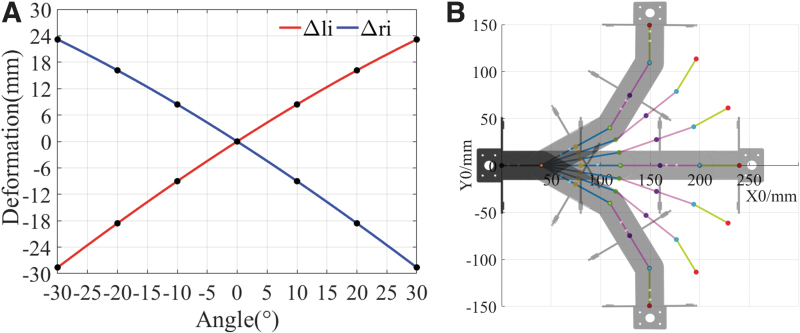
**(A)** Relationship between the deflection angle of each submodule of the flexible spine and the corresponding shape variable of the SMA spring. **(B)** The posture of each position during the flexible spine deflection process.

The relationship between the shape variable and the deflection angle of the module is shown in Figure 5A. Denavit–Hartenberg parameters were obtained from the frame model of the flexible spine to describe the orientation of the flexible spine's head *C*_4_ relative to its tail *C*_1_. According to the parameters of the connecting rods and the joints, the transformation matrices of each connecting rod were calculated and the final homogeneous transformation matrix can be given as follows:

(5) $$T=\left[\begin{array}{cccc} C\left(\theta_{1}+\theta_{2}+\theta_{3}\right) & -S\left(\theta_{1}+\theta_{2}+\theta_{3}\right) & 0 & 2nC\left(\theta_{1}+\theta_{2}\right)+2nC\left(\theta_{1}\right)+n\\ S\left(\theta_{1}+\theta_{2}+\theta_{3}\right) & C\left(\theta_{1}+\theta_{2}+\theta_{3}\right) & 0 & 2nS\left(\theta_{1}+\theta_{2}\right)+2nS\left(\theta_{1}\right)\\ 0 & 0 & 1 & 0\\ 0 & 0 & 0 & 1 \end{array}\right],$$

where *C* denotes cos and *S* represents sin. Here, *O*_0_ was used as the origin, and $X_{0}-Y_{0}$ was taken as the plane coordinate system. The coordinates of *C*_4_ in this plane can be expressed as follows:

(6) $$\left\{\begin{array}{c} x_{0}^{C_{4}}=n+2nC\left(\theta_{1}\right)+2nC\left(\theta_{1}+\theta_{2}\right)+nC\left(\theta_{1}+\theta_{2}+\theta_{3}\right)\\ y_{0}^{C_{4}}=2nS\left(\theta_{1}\right)+2nS\left(\theta_{1}+\theta_{2}\right)+nS\left(\theta_{1}+\theta_{2}+\theta_{3}\right) \end{array}\right.$$

In this work, the SMA springs on each side were in series for convenient control. In this situation, the contraction shape variable generated by the SMA spring in each submodule will be consistent, which implies that $\theta_{1}=\theta_{2}=\theta_{3}$. Figure 5B illustrates the posture of each position during deflection of the flexible spine.

### Collaborative model of flexible spine and limbs

**FIG. 6.**
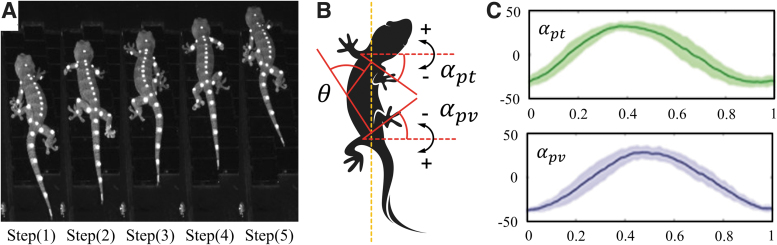
**(A)** Posture and lateral trunk swing of a gecko during one gait cycle. **(B)** Definitions of the pectoral girdle angle $\alpha_{pt}$ and pelvic girdle angle $\alpha_{pv}$ in the gecko trunk. **(C)** Changes in pectoral girdle/pelvic girdle angle during one gait cycle.^39^

At present, the morphological and kinematic characteristics of quadrupedal vertebrates with flexible trunks have been extensively examined in the literature.^8–10^ Taking the gecko as an example, Figure 6A shows the posture of the gecko during a trot gait cycle. According to Wang's research, the maximum unilateral fluctuation ranges of the pectoral girdle angle $\alpha_{pt}$ and pelvic girdle angle $\alpha_{pv}$ of the gecko trunk are 33.56 ± 3.01° and 33.22 ± 1.72°, respectively, during one gait cycle (Fig. 6B, C).^39^

Based on the observations of gecko morphology and kinematics, four assumptions were proposed to simplify the theoretical analysis: (1) When the trunk swings laterally, the rotations of the pectoral and pelvic girdles are symmetrical in time and space. (2) At the moment of gait transition, the changes in the angles of the corresponding limb joints are the same. (3) The rotation direction and degree of each rotating joint of the flexible spine are the same. (4) The influence of the tail on movement can be ignored.

**FIG. 7.**
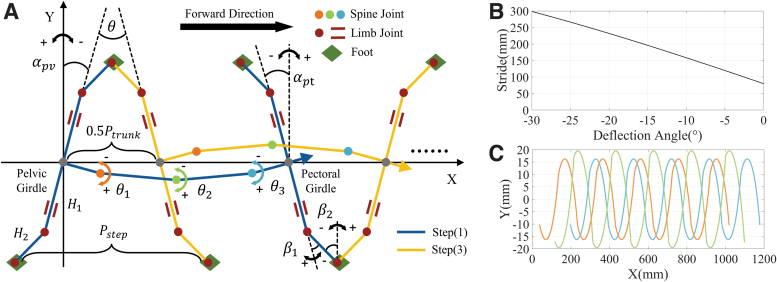
**(A)** Cooperative motion model of the flexible spine and limbs of the gecko-like robot. **(B)** Relationship between the motion stride and the initial angle of the pectoral/pelvic girdles. When $\alpha^{\circ }=0^{\circ }$, the stride is 80.0 mm; when $\alpha^{\circ }=-15^{\circ }$, the stride is 196.0 mm, and when $\alpha^{\circ }=-30^{\circ }$, the stride is 298.6 mm. **(C)** Trajectories of each rotating joint of the flexible spine in the coordinate plane. The maximum periodic lateral displacements of the three joints relative to the direction of motion were ±16.26, ±19.54, and ±16.26 mm, respectively.


According to the abovementioned assumptions, a collaborative model of the flexible spine and limbs was established, as shown in Figure 7A. One gait cycle *S* is defined as the time that the right hind foot contacts the motion plane two consecutive times. The stride $P_{step}$ can be defined as the distance between adjacent footholds of each leg. The angles in the model can be related by the following equation:

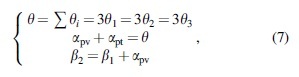


where θ denotes the deflection angle of the spine, $\beta_{1}$ is the knee joint variable, $\beta_{2}$ represents the ankle joint variable, and $\alpha_{pt}$ and $\alpha_{pv}$ are equal to α.

The orientation of the feet was assumed to be constant. Furthermore, the hip joint was set as a pitching joint, which was not a variable factor in the lateral swing of the trunk. The stride and movement distance during one cycle can be expressed as follows:

(8) Pstep=Ptrunk=4(H1 sinα0+H2 sinβ20),

where $P_{trunk}$ denotes the straight-line distance that the robot moves in one cycle, *H*_1_ stands for the length from the knee to the center of the pelvic girdle, *H*_2_ represents the length from the ankle to the knee, and $\alpha^{0}$ and $\beta_{2}^{0}$ refer to the initial angle of the pectoral/pelvic girdle and the ankle, respectively. As can be observed from Equations (7) to (8), the stride depends on the initial angle of the pectoral girdle when the knee joint variable is fixed. The relationship between the two is shown in Figure 7B. Here, $H_{1}=80$ mm and $H_{2}=40$ mm. The range of $\alpha^{0}$ was set to −30° to 0°. It can be seen that the stride increased very obviously with the increase in the deflection angle. However, the collision of ipsilateral legs and the instability of the robot centroid will be induced if the pectoral girdle/pelvic girdle angle is too large.^40^

Under comprehensive consideration, $\alpha^{0}$ = −15° was chosen as a compromise. The initial rotation angle of the knee joint $\beta_{1}^{0}$ was set to −30°, while $\beta_{2}^{0}$ was set to −45°. One gait cycle was set as *S* = 4 s. The changes in $\beta_{1},\beta_{2}$ and α with *t* can be expressed as follows:

(9) $$\beta_{1}=\left\{\begin{array}{c} -30+30\left(t-4k\right),4k\leq t<4k+2\\ 90-30\left(t-4k\right),4k+2\leq t<4\left(k+1\right) \end{array}\left(k=0,1,2,\ldots \right)\right.,$$

(10) $$\beta_{2}=\left\{\begin{array}{c} -45+45\left(t-4k\right),4k\leq t<4k+2\\ 135-45\left(t-4k\right),4k+2\leq t<4\left(k+1\right) \end{array}\left(k=0,1,2,\ldots \right)\right.,$$

(11) $$\alpha =\left\{\begin{array}{c} -15+15\left(t-4k\right),4k\leq t<4k+2\\ 45-15\left(t-4k\right),4k+2\leq t<4\left(k+1\right) \end{array}\left(k=0,1,2,\ldots \right)\right.$$

In the collaborative model, the lateral swing of the spine mainly occurs in the form of a standing wave. The plane coordinate changes of the three rotating joints can be expressed as follows:

(12) $$\left\{\begin{array}{c} x_{\theta_{1}}=x_{pv}+nC\left(\alpha \right)\\ y_{\theta_{1}}=y_{pv}+nS\left(\alpha \right)\\ x_{\theta_{2}}=x_{pv}+nC\left(\alpha \right)+2nC\left(\alpha -\theta_{1}\right)\\ y_{\theta_{2}}=y_{pv}+nS\left(\alpha \right)+2nS\left(\alpha -\theta_{1}\right)\\ x_{\theta_{3}}=x_{pv}+nC\left(\alpha \right)+2nC\left(\alpha -\theta_{1}\right)+2nC\left(\alpha -\theta_{1}-\theta_{2}\right)\\ y_{\theta_{3}}=y_{pv}+nS\left(\alpha \right)+2nS\left(\alpha -\theta_{1}\right)+2nS\left(\alpha -\theta_{1}-\theta_{2}\right) \end{array}\right.,$$

where $\left(x_{pv},y_{pv}\right)$ denotes the coordinates of the center of the pelvic girdle, which can be determined by using the following sets of equations:

(13) xpv=xlh+kPstep+H1 sinα+H2 sinβ2,4k≤t<4k+2xlh+2k+12Pstep−H1 sinα−H2 sinβ2,4k+2≤t<4k+1k=0,1,2⋯,

(14) ypv=ylh−H1 cosα−H2 cosβ2,4k≤t<4k+2−ylh+H1 cosα+H2 cosβ2,4k+2≤t<4k+1k=0,1,2⋯,

where $\left(x_{lh},y_{lh}\right)$ denote the foothold coordinates of the left hind leg in the initial state of the robot, which can be expressed as follows:

(15) xlh=H1 sinα0+H2 sinβ20ylh=H1 cosα0+H2 cosβ20

Here, *n* = 40 mm was set in the structural design. The theoretical moving speed of the robot was 48.73 mm/s. The motion trajectory of each rotating joint in the coordinate plane can be drawn, as shown in Figure 7C. From the extracted outcomes, it was found that the trajectory of each rotating joint of the spine has the same trend of change. On top of that, in the upper and lower parts of a gait cycle, the same joint presents a symmetrical change trend in time.

## Experimental Validation and Discussion

### Experimental preparation

The flexible spine and limb mechanisms of the robot were made of resin material by using 3D printing technology. The head and tail were cut and processed from a 2-mm-thick carbon fiber plate. The overall mass of the robot was 1.03 kg and its length and width were 970 and 162 mm, respectively.

**FIG. 8.**
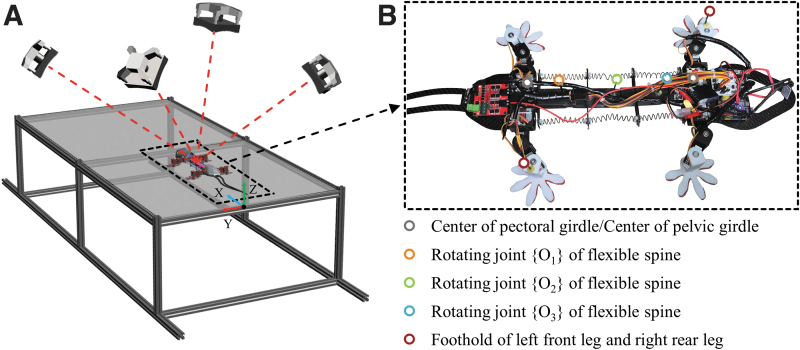
Kinematic experiment system. **(A)** The platform comprised an aluminum alloy frame and a plate made of transparent acrylic with dimensions of 1.80 × 0.86 m. Four high-speed cameras (Prime, 17w; NaturalPoint, Inc., Corvallis, OR) were distributed around the platform, all of which had their own field of view. The overlapping field of view area constituted the motion range of the robot. The cameras were triggered synchronously at a shooting frequency of 60 Hz. An LED lamp around the lens projected 850 nm near-infrared light, which was received by the camera after being reflected by the marker *dots*. **(B)** The marked *points* included the center of the pectoral/pelvic girdle, three rotating joints of the flexible spine, and the footholds of the left front leg and right rear leg. The frame *O*, *X*, *Y*, *Z* was fixed to the earth.

To determine whether the gecko-like robot meets the theoretical expectations, a series of experiments were carried out. Each body feature was marked with fluorescent dots. Next, the robot was placed on a motion platform, as can be observed from Figure 8. This experimental system determined the 3D coordinates of the marked points in the whole spatial area.^39^ Then, the absolute coordinates of these points at any time were obtained by using the direct linear transformation method (HeDrik Lab, University of North Carolina), and the motion trajectories of all marked points were simulated accordingly.^41,42^

### Straight motion experiment

In the experiment, the springs were preheated before the robot started moving. During the preparation phase, the frequency of the PWM signals was set to the value of 20 Hz and the duty cycle was adjusted to 30%. The temperature of the SMA springs increased slowly, but not enough to cause deformation. In the initial state, the duty cycles of the left and right PWM signals were set, respectively, to 10% and 50% to make the spine swing laterally to the right. At this time, the left foreleg and the right hind leg of the robot stepped forward, while the right foreleg and the left hind leg stepped backward. When the robot started to move, the duty cycles of the left and right PWM signals were adjusted to 50% and 10%, respectively, to make the spine swing to the left.

**FIG. 9.**
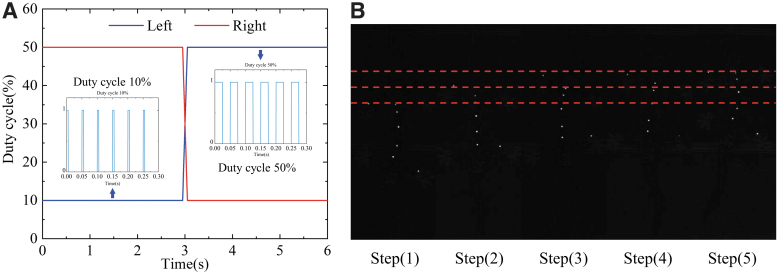
**(A)** Variations in the PWM control signals on each side of the flexible spine during a gait cycle. **(B)** The corresponding posture of the bionic gecko robot during a gait cycle. The *red dotted lines* mark the positions of the pectoral girdle center at the beginning, middle, and end of a cycle.

During this process, the robot lifted the right foreleg and the left hind leg and extended them forward, while the left foreleg and the right hind leg swung backward relative to the trunk. During the following movement, the PWM signals were adjusted symmetrically, while the limbs made the corresponding swings. The robot moved forward periodically through the control method described above. See Supplementary Videos S1–S4 for the motion results of the robot. Figure 9 illustrates the changes in the PWM control signals on both sides and the corresponding posture of the robot over one cycle. Due to the limitations of the servo motor speed and the heating/cooling frequency of the SMA spring, the duration of one cycle was *S* = 6 s.

**FIG. 10.**
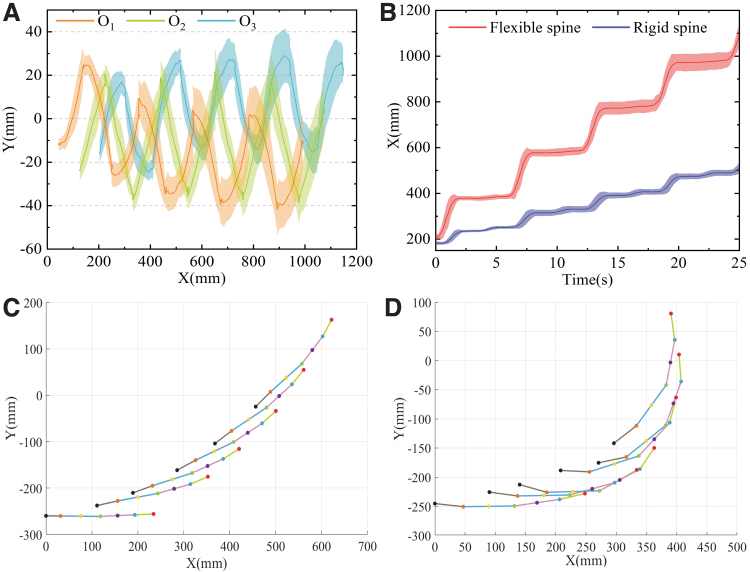
Experimental results. **(A)** Movement tracks of each joint of the flexible spine that was recorded by cameras. **(B)** Comparison of the stride of the robot with a rigid and flexible spine. **(C)** Movement trajectory of each point on a rigid spine and **(D)** movement trajectory of each point on a flexible spine (connecting *lines* represent the spine).

A total number of 10 straight motion tests were conducted. In each test, the robot accomplished four complete gait cycles in the crawling plane. To visualize this, the data recorded by the cameras were plotted as a curve. Figure 10A depicts the motion trajectories of the three rotation joints of the spine. From the acquired results, it can be argued that: (1) Generally speaking, each spine joint tends to oscillate back and forth along the *y*-axis direction. In other words, the body always shows the form of a standing wave, which is highly consistent with the theoretical model.

(2) Unfortunately, from the fluctuating trajectories of *O*_1_, *O*_2_, and *O*_3_, it was proven that the average difference between the peak and trough of each joint was 38.29, 45.81, and 39.45 mm, respectively. These values are different from the theoretical values, representing errors of 17.74%, 21.31%, and 17.22%, respectively. Furthermore, it was found that these errors increase gradually with the progress of the robot's motion. This may be related to the hysteresis of the SMA springs on each side. In short, when the spring on the stretched side is not completely cooled and is then heated again in the next round, its temperature becomes bigger despite receiving the same power, which leads to a gradual increase in its contraction shape variable. Consequently, the torsion of the spine is increased and the error described above occurs.

(3) The joint trajectory shows that the robot moved forward about 850 mm over four gait cycles. Note that during the movement of the robot, the joint *O*_1_ gradually shifted along the *y*-direction and the joint *O*_3_ gradually shifted along the *y-*direction, with offsets of 20.41 and 9.86 mm, respectively. This effect also led to a larger oscillation displacement of joint *O*_2_ in the *y*-direction. On the whole, this means that the orientation of the robot is gradually biased to the left. The reason is that its initial state is with the spine twisted to the right and the head tilted to the left. In the subsequent movement, due to some relative sliding and rotation between the sole of the foot and the plane, the robot could not tilt its head to the right completely symmetrically. This behavioral error is accumulated with each cycle.

In general, the collected experimental results verify the cooperative motion model of the flexible spine and limbs.

To show the contribution of the flexible spine to the stride motion, stride motions with and without lateral swing (flexible or rigid spine) were compared. Similarly, the moving tracks of the footholds of the limbs along the forward direction were recorded, as can be seen from Figure 10B. By considering that the swing of each leg was the same in a gait cycle, the left foreleg was selected to be recorded here. From the experimental data, it can be argued that when the robot moved with a rigid spine, its average stride was 81.63 mm and its moving speed was $v_{r}=20.35$ mm/s. On the contrary, when the flexible spine was used, the average stride was 199.88 mm and the moving speed was $v_{s}=44.89$ mm/s. This result is in direct line with our theoretical expectations and confirms that lateral trunk swing has a very positive impact on the stride motion.

### Turning motion experiment

To test the actual turning ability of the robot prototype, turning experiments were also carried out on the same motion platform. Taking a left-turn motion as an example, when the robot adopted a rigid trunk, the rotating joints on the spine were limited. In contrast, when the flexible trunk was used, the restriction of the rotating joint was lifted. The duty cycle of the electric heating signal of the left SMA spring was adjusted to 80%, while the limb movement remained unchanged. The robot's motion is represented as a moving track of each point on the spine in Figure 10C and D. Through the calculations, the turning radius of the robot with the rigid spine was about 620 mm and that with the flexible spine was about 270 mm. Hence, the flexible spine greatly reduced the turning radius of the robot.

By controlling the degree of spring contraction on the heated side, which is changing the spine deflection angle, the robot can perform a turning motion when 270 mm ≤r≤ 620 mm (*r* = the turning radius), which can greatly improve the flexibility of the robot.

## Conclusion

The bendable trunk of a gecko enables it to trot with a lateral swing pattern, by increasing the stride and movement speed. At the same time, a flexible spine provides greater turning agility. In this work, a gecko-like robot with a flexible spine based on SMA springs was proposed. The driving tension and deformation displacement of the SMA springs were measured experimentally, and the static parameters of the SMA spring were obtained. Based on the acquired results, a kinematic model of a flexible spine was established and the mathematical relationship between the deflection angle of the flexible spine and the contraction deformation of the SMA springs was systematically analyzed.

Furthermore, a collaborative motion model of a flexible spine and limbs was established to describe the specific relationships between leg joint variables and spine deflection angle during the trot gait with a lateral swing pattern. Therefore, a robust theoretical reference for the formulation of robot motion strategies was provided. A prototype gecko-like robot was also fabricated based on the theoretical model, and straight-line and turning experiments were carried out. From the experimental results, it was demonstrated that the stride of a robot with a rigid spine was 81.63 mm and that with a flexible spine was 199.88 mm. The lateral swing of the trunk greatly improved the stride of the robot. The collaborative motion model was also verified by the conducted experiments. Compared with a rigid trunk, the turning radius of a robot with a flexible spine was greatly reduced, giving the robot more flexible movement.

In the future, gecko-like robots with flexible trunks will be further developed. Here, the method of installing adhesive materials on the foot of the robot was adopted. As a result, the robot exhibited a certain climbing ability, whereas the maximum angle that the robot can climb was about 25°. Nevertheless, the stability of the robot cannot be guaranteed during the movement. In the next stage, more attention should be paid to the foot adhesion mechanism for the development of robots with even better agility. At the same time, the feedback compensation will be increased based on the open-loop electric heating control of the SMA springs. This is expected to further reduce the nonlinear hysteresis impact of the SMA spring and improve the positional accuracy of the flexible spine deflection.

## Supplementary Material

Supplemental data

Supplemental data

Supplemental data

Supplemental data
